# Coping moderates the relationship between intolerance of uncertainty and stress in men during the Covid-19 pandemic

**DOI:** 10.1590/1980-220X-REEUSP-2021-0303

**Published:** 2022-01-07

**Authors:** Emanuel Missias Silva Palma, Anderson Reis de Sousa, Franciane Andrade de Morais, Ramon Evangelista Luz, Álvaro Lima Freitas, Pâmela Pitágoras Freitas Lima

**Affiliations:** 1 Escola Bahiana de Medicina e Saúde Pública, Salvador, BA, Brazil.; 2 Universidade Federal da Bahia, Escola de Enfermagem, Pós-Graduação em Enfermagem e Saúde, Salvador, BA, Brazil.; 3 Instituto Federal Baiano, Salvador, BA, Brazil.; 4 Faculdade Unidas de Pesquisas, Ciências e Saúde, Jequié, BA, Brazil.; 5 Fundação Santo André, Santo André, SP, Brazil.

**Keywords:** Men’s health, Mental health, Psychological adaption, Psychological stress, Covid-19, Salud del hombre, Salud mental, Adaptación Psicológica, Estrés Psicológico; Covid-19, Saúde do homen, Saúde mental, Adaptação psicológica, Estresse psicológico, Covid-19

## Abstract

**Objective:**

To test the explanatory power of coping strategies and intolerance of uncertainty on men’s perceived stress levels and test the moderating role of coping strategies in the relationship between intolerance of uncertainty and perceived stress during the Covid-19 pandemic.

**Method:**

This was an online cross-sectional study in which 1,006 men living in Brazil during the Covid-19 pandemic participated. Participants were recruited using a snowball sampling technique and completed a questionnaire containing measures of all study variables. Data were examined using a correlation and a regression analysis.

**Results:**

Intolerance of uncertainty (β = .51) and refusal (β = .15) positively predicted perceived stress, whereas control (β = –.31) and isolation (β = –.06) negatively predicted it. Together, these variables explained 52% of men’s perceived stress (*p* < .001). Isolation and social support lessened the relationship between intolerance of uncertainty and stress (p < .001).

**Conclusion:**

Men high in intolerance of uncertainty and refusal were more vulnerable to stress during the pandemic. However, coping helped mitigate the relationship between intolerance of uncertainty and perceived stress, thus being a promising psychosocial intervention in this context.

## RESUMEN

**Objetivo:**

Testar el poder explicativo de las estrategias de enfrentamiento e intolerancia a la incertidumbre en los niveles de estrés notados por los hombres y testar el rol moderador de las estrategias de enfrentamiento en la relación entre la intolerancia a la incertidumbre y el estrés notado durante la pandemia de Covid-19.

**Método:**

Estudio transversal online del cual participaron 1.006 hombres que vivían en Brasil durante la pandemia de Covid-19. Los participantes fueron seleccionados por medio de una técnica de muestreo en bola de nieve y rellenaron un cuestionario conteniendo medidas de todas las variables del estudio. Los datos fueron examinados utilizando correlación y análisis de regresión.

**Resultados:**

Intolerancia a la incertidumbre (β = 0,51) y rechazo (β = 0,.15) hicieron un pronóstico positivo al estrés notado, mientras el control (β = −0,31) y el aislamiento (β = −0,06) lo pronosticaron negativamente. Juntas, esas variables explicaron 52% del estrés percibido por los hombres (*p*< 0,001). El aislamiento y el apoyo social disminuyeron la relación entre la intolerancia a la incertidumbre y el estrés (p < .001).

**Conclusión:**

Hombres con alta intolerancia a la incertidumbre y rechazo eran más vulnerables al estrés durante la pandemia. Sin embargo, el enfrentamiento ayudó a amenizar la relación entre la intolerancia a la incertidumbre y el estrés notado, por lo que fue una intervención psicosocial promisora en ese contexto.

## RESUMO

**Objetivo:**

Testar o poder explicativo das estratégias de enfrentamento e intolerância à incerteza nos níveis de estresse percebidos pelos homens e testar o papel moderador das estratégias de enfrentamento na relação entre a intolerância à incerteza e o estresse percebido durante a pandemia de Covid-19.

**Método:**

Estudo transversal online do qual participaram 1.006 homens que moravam no Brasil durante a pandemia de Covid-19. Os participantes foram recrutados por meio de uma técnica de amostragem em bola de neve e preencheram um questionário contendo medidas de todas as variáveis do estudo. Os dados foram examinados usando uma correlação e uma análise de regressão.

**Resultados:**

Intolerância à incerteza (β = 0,51) e recusa (β = 0,.15) previram positivamente o estresse percebido, enquanto o controle (β = −0,31) e o isolamento (β = –0,06) previram-no negativamente. Juntas, essas variáveis explicaram 52% do estresse percebido pelos homens (*p*< 0,001). O isolamento e o apoio social diminuíram a relação entre a intolerância à incerteza e o estresse (p < .001).

**Conclusão:**

Homens com alta intolerância à incerteza e recusa eram mais vulneráveis ao estresse durante a pandemia. No entanto, o enfrentamento ajudou a amenizar a relação entre a intolerância à incerteza e o estresse percebido, sendo uma intervenção psicossocial promissora nesse contexto.

## INTRODUCTION

Higher rates of mental health disorders have been reported worldwide during the months following the Covid-19 pandemic^(1)^, posing challenges to health professionals of different fields, such as psychology and nursing. In this context, individuals were more likely to report greater levels of depression, anxiety, insomnia, and somatic disorders^(2)^. Studies have been carried out in different populations, such as health care professionals^(3,4)^, women^(5)^, and adolescents^(6)^. However, little attention has been paid to men’s mental health issues in nursing professional practice^(7)^, even though men were more likely to be contaminated by the SARS-CoV-2^(7)^ and to commit suicide^(8)^ in this context. In recent months, a surge of interest in mental health indicators, such as stress and coping, has been observed in the literature.

Stress has traditionally been defined as an intense experience of strain involving physical, emotional, and cognitive dimensions^(9)^. Perceived stress (PS) has consistently been linked to adverse mental and physical health outcomes, such as cardiovascular disease, burnout syndrome, insomnia, and fatigue^(10)^. In addition, some events have been significant antecedents of the stress response in humans (i.e., stressors), such as unexpected environmental changes, deaths, and lower socioeconomic status^(11)^. Furthermore, intraindividual variables have been found to either increase or decrease individuals’ PS levels. This study focuses on two of these variables, namely intolerance of uncertainty (IU) and coping.

Intolerance of uncertainty (IU) has been referred to as the tendency to respond or react negatively to ambiguous contexts^(12)^. These authors also hold that IU is a dispositional trait and a transdiagnostic factor common to a host of psychological disorders. IU has consistently been linked to depressive and anxiety symptoms in different populations and age groups^(12)^. Some argue that contexts involving unpredictable events, ambiguous information, and lack of control might increase an individual’s level of IU, leading to greater psychological distress and other adverse mental health outcomes^(13)^. Pandemic contexts, such as the one currently experienced by the world population to different degrees, contain those elements associated with higher IU^(14)^. In this sense, researchers have focused their attention on other individual resources that might either increase or attenuate the effects of IU on an individual’s mental health outcomes. In this context, coping strategies might be seriously considered because they might function as either protective or risk factors at an individual level.

Coping can be defined as a dynamic and contextualized individual response to psychological and environmental stress^(15)^. From this perspective, coping should not always be associated with positive health outcomes, and researchers must consider the demands posed by stressful events^(16)^. In line with this argument, a model of coping integrating strategies into four categories was proposed^(16)^, namely (a) control, which refers to regulating emotional and behavioral reactions to the problem situation through active problem solving; b) social support, which involves seeking others’ instrumental, informational, and emotional aid; c) refusal, which entails avoiding daily activities and social interactions either through fantasy, escape, or distraction from aversive situations; and d) isolation, which refers to behavioral change to adapt to the context even when it involves keeping oneself apart from others^(16)^. For example, positive coping strategies (e.g., seeking help and active problem solving) were associated with lower emotional distress, whereas negative coping increased psychological suffering during the Covid-19 pandemic in China^(17)^. Similarly, it was found that American and Canadian individuals with anxiety and mood symptoms related to the Covid-19 pandemic were more likely to experience greater stress and respond to it with self-isolation strategies than those with no psychological disorder^(18)^. These findings highlight that intraindividual characteristics, such as IU and coping strategies, might influence individual stress responses.

As observed, these findings considerably affect nursing practice in its different dimensions, such as knowledge production, clinical practice, and care management. Moreover, the constructs under investigation are part of the functional domains of nursing; therefore, understanding their mechanisms and effects on health might help strengthen nursing practice and science at a global level.

Considering the literature reviewed, the objective of this study was to test the explanatory power of coping strategies and IU on men’s PS levels and test the moderating role of coping strategies in the relationship between IU and PS in men during the Covid-19 pandemic. It was hypothesized that higher levels of IU would be associated with greater PS levels and that control and social support coping strategies would be associated with lower PS levels. In addition, refusal and isolation strategies were expected to be related to greater PS. Finally, it was hypothesized that the coping strategies used by men would moderate the relationship between IU and PS.

## METHOD

### Design of Study

This was a cross-sectional online survey carried out nationwide in Brazil. The larger project of which the present study is part is entitled Saúde mental de homens na pandemia do novo coronavirus in Brazil and is coordinated by the Grupo de Estudos sobre o Cuidado em Saúde (GECS) at the Escola de Enfermagem da Universidade Federal da Bahia (UFBA). The guidelines suggested by the Strengthening the Reporting of Observational Studies in Epidemiology (STROBE) were followed.

### Population and Sample

Participants in the study were 1,006 men living in Brazil at the time of data collection. Most self-identified as non- heterosexual (54.1%), “pardo” (brown-skinned) (39.5%) and aged between 29 and 39 (45.1%) with a college degree (73.8%). Most had a paid job (75%) and earned up to two minimum Brazilian wages (41.6%). A minimum sample of 923 participants was estimated, adopting as parameters the population (N) of 69,324,099 Brazilian men with Internet access^(20)^, an expected proportion of 50% for the event of interest, a confidence level of 95%, 5% accuracy, 80% power, design effect equal to 2, and 20% increase for dropouts or problems with the Internet at the time of questionnaire completing.

The inclusion criteria were being self-declared men of 18 years or older and living in Brazil during the months following the Covid-19 pandemic. Therefore, women and men under 18 were excluded from the study sample.

### Data Collection

Data were collected between April and June 2020, period when cases of Covid-19 were increasing in Brazil. Furthermore, some sanitary control measures for containing the disease were used (e.g., quarantine, physical distancing, and mask-wearing). Data were collected nationwide through an anonymous online questionnaire using a snowball sampling technique.

The sample was selected using the methodological criteria of the technique of consecutive recruitment of participants. Simultaneous, not sequential, selection strategies were adopted in the five Brazilian regions. Five initial seeds were defined based on access to 20 possible male participants, with unique characteristics among them, namely: geographic location/region of the country, state, and area of residence (rural or urban); race/color (e.g., white and non-white); age (not elderly and elderly); education level (e.g., high school and college level). Access to these participants took place in the virtual environment of digital social networks through groups, pages, and communities on Facebook, Instagram, and Twitter related to the theme of male health and the Covid-19 pandemic in the country’s five regions. Each participant invited received the survey link and was guided and encouraged to invite other survey participants from their social network to ensure continued recruitment and obtain a meaningful sample.

Because the present survey was conducted in the virtual environment, it was possible to measure 27 last seeds, corresponding to the number of Brazilian states represented in the study. This control was based on geolocation features, through direct chat with online users, monitoring the drive and engagement on Facebook and Instagram social networks, from fixed posts on the official research page (@caredesaudedehomens) and the responsible team. Therefore, to ensure accuracy, quality, excellence, reliability, and transparency in the development and presentation of the study, the recommendations proposed in the Standards Quality Improvement Reporting Excellence (SQUIRE 2.0) were adopted.

### Instruments

Perceived Stress Scale (PSS)^(21–27)^: This is a 14-item instrument using a Likert-type scale ranging from 0 = never to 4 = always. Respondents indicate how often they have felt or done something over the last four weeks (ex.: *In the previous month, how often have you felt you were unable to control important things in your life/felt nervous or stressed?*). A study was conducted to test its psychometric properties in a Brazilian sample and found good validity and reliability evidence^(21)^. In the present study, the internal reliability of the PSS as assessed by the Cronbach alpha was α = .88.

Intolerance of Uncertainty Scale (IUS–12)^(11)^: This is the adapted short version of the IUS original scale^(12)^ and comprises 12 items. It is rated on a 5-point Likert scale (1 = not all characteristic of me; 5 = entirely characteristic of me). An example item is *It frustrates me not having all the information I need*. It was validated in Brazil and showed good psychometric properties^(22)^. In the present study, the IUS-12 demonstrated excellent internal consistency (α = .89).

Toulousaine Coping Scale Shortened Version:^(19,23)^ This is an 18-item using a 5-point Likert-type instrument. Respondents indicate how often (1 = never and 5 = very frequently) they use several coping strategies to deal with stressful situations. The scale consists of four dimensions with an example item and their respective indexes of internal reliability (Cronbach alpha) in the present study, namely: control (e. g.: *I face the situation*.; α = .77); social support (e.g., *I seek help from friends to relieve my anxiety*; α = .60); refusal (e.g., *I try not to think about the problem*.; α = .61), and isolation (e.g., *I avoid meeting people*.; α€= 61). It has shown good psychometric properties in validation studies in Brazil^(24,25)^.

Sociodemographic questionnaire: This contained men’s sexual identity, occupation, educational level, age, and monthly earnings.

### Data Analysis

First, a correlation analysis was used to assess the associations among all study variables. Then, a regression analysis using the enter method was carried out using a bootstrapping procedure with 1000 bootstrap samples and bias-corrected accelerated 95% confidence intervals. Finally, a regression analysis using the Process Macro^(26)^ was carried out to test the moderating effects of the four coping strategies in the relationship between total IU and PS.

### Ethical Aspects

The Institutional Review Board approved the larger project of which this study is a part at n. 4.087.611. All the guidelines laid down by the Brazilian National Health Council (CNS 466/2012) were followed. Informed consent was obtained before data collection by clicking on the appropriate box in the online questionnaire.

## RESULTS

There were weak to strong correlations between PS and all other study variables, except isolation. For example, higher levels of IU and refusal were associated with greater PS. In contrast, higher levels of control and social support were associated with less PS. Table 1 shows the bivariate correlations among all study variables.

**Table 1.** Means, Standard Deviations and Correlations among Study Variables – Brazil, 2020.

		M	SD	1	2	3	4	5	6
1.	PS	27.28	9.44	–					
2.	IU	3.04	.85	.63***	–				
3.	Control	4.00	.72	–.47 ***	–.25***	–			
4.	Isolation	2.72	.73	.03	.21***	.11***	–		
5.	Refusal	3.01	1.05	.34***	.36***	–.04***	.14***	–	
6.	Social support	3.61	.96	–.28***	–.12***	.52***	.09***	–.14***	–

*Note.* PS = Perceived Stress; IU = Intolerance of Uncertainty ***p < .001

In multiple regression analysis, moderate to strong significant associations were found between the criterion variable, PS, and all predictor variables, except social support. Together, IU, control, isolation, and refusal explained 52% of the variance in PS scores. Both IU and refusal were positively related to PS, whereas control and isolation were negatively associated with it. Table2 shows the standardized regression coefficients for all predictor variables. It also reveals that IU was the strongest positive predictor of PS, whereas control was its strongest negative predictor.

**Table 2.** Regression Coefficients for Perceived Stress – Brazil, 2020.

Variable	***B***	95% CI for B		*SE B*	β	*R* ^2^	∆*R* ^2^
		*LL*	*UL*				
						.52	.52***
Constant	1.85	1.62	2.10	.12			
IU	.40	.36	.44	.02	.51***		
Control	–.30	–.35	–.24	.03	–.31***		
Isolation	–.06	.01	–.09	–.01	–.06***		
Refusal	.09	.06	.13	.02	.15***		
Social Support	–.02	–.06	.02	.02	–.03		

*Note.* CI = confidence interval; LL = lower limit; UL = upper limit ***p < .001

**Figure 1.**
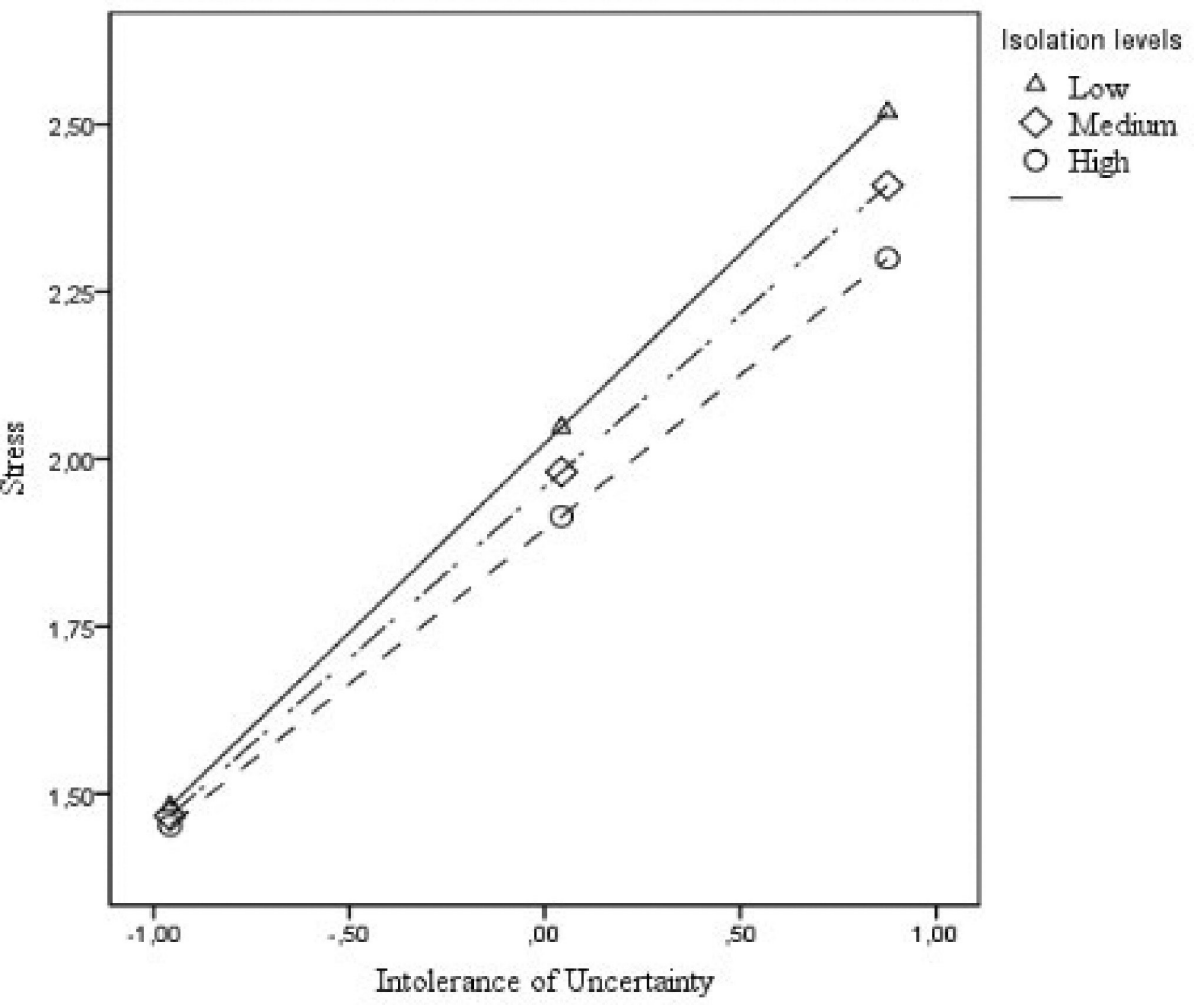
Isolation moderates the effects of IU on PS.

**Figure 2.**
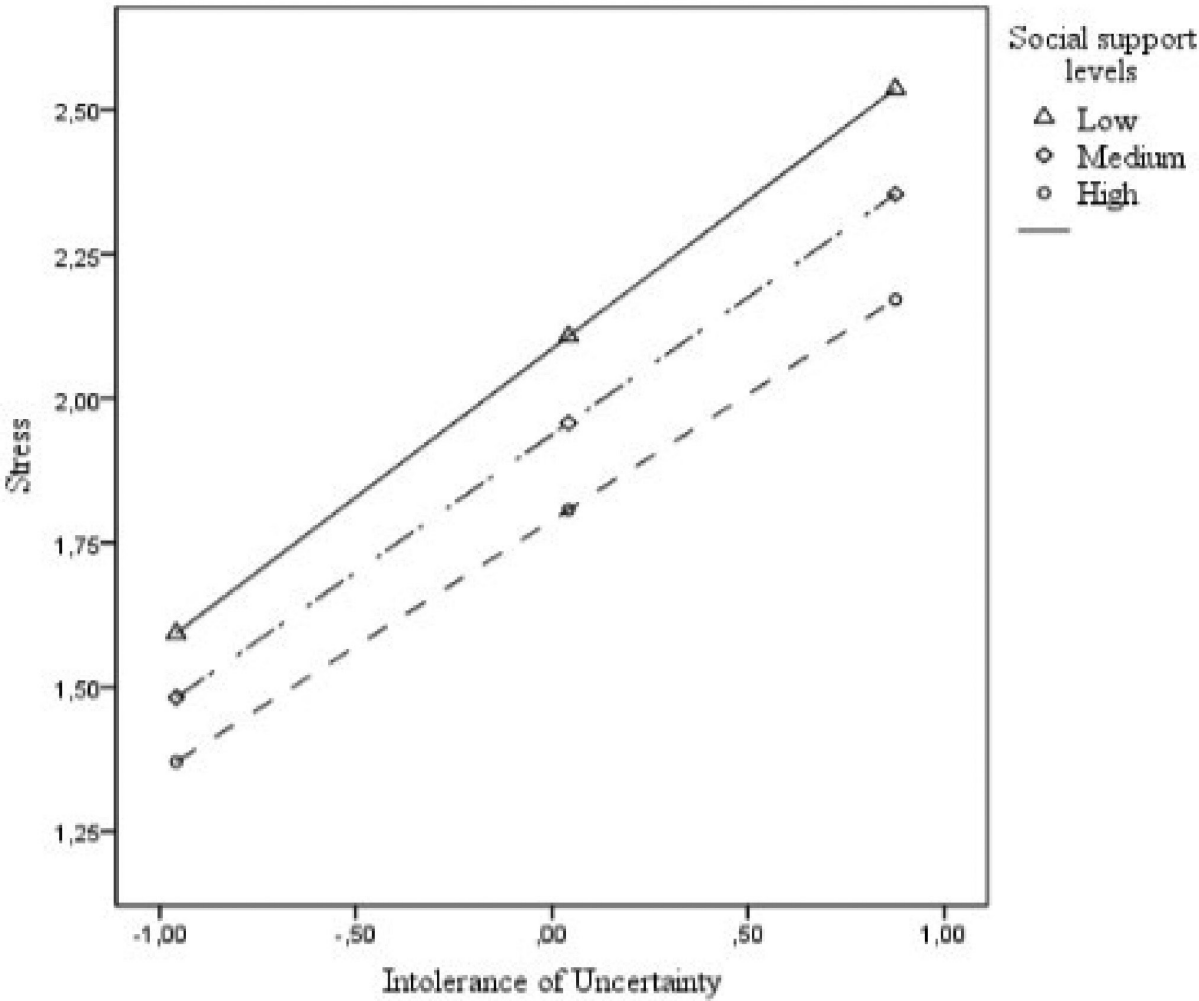
Social support moderates the effects of IU on PS.

A moderation analysis using the Process Macro was carried out to test how coping strategies would moderate the relationship between IU and PS. Statistically significant effects were found for isolation *F*(1, 1.006) = 10.00, *p* = .001, and social support *F*(1, 1.006) = 4.24, *p* = .04. When a significant moderation was found, the moderating variable was divided into three parts for better visualization of the interaction, adopting the following cut-off points: lower 16%, median 64%, and higher 16%. Figures 1 and 2 facilitate the visualization of the moderating effects of isolation and social support on the relationship between IU and PS. As seen, both variables lessen the effects of IU on PS. In other words, the relationship between IU and PS was lowest when men reported high levels of either isolation (Figure 1) or social support (Figure2).

## DISCUSSION

This study’s main objective was to test the explanatory power of IU and coping strategies on men’s PS levels and test the moderating role of coping strategies in the relationship between IU and PS. The findings partially corroborated the study hypotheses.

In terms of the predictive model, as expected, IU predicted higher levels of men’s PS. This finding reinforces IU’s role as a transdiagnostic factor associated with adverse mental health outcomes, as reported by other studies^(12,13,22)^. The present study findings help to highlight that IU, understood as an intraindividual characteristic triggered or aggravated by an adverse context, increases the likelihood of psychological suffering. This relationship has substantial implications for nursing and health care practice during the pandemic. For instance, treatment and intervention studies might focus on dampening this deleterious association, an issue the present study empirically addressed by testing a moderating model in which coping strategies were used as moderators. Therefore, deepening disciplinary knowledge in nursing and health can uniquely contribute, broadening our understanding of stress and its subtypes, such as acute stress, chronic stress, and post-traumatic stress disorders. Additionally, these findings might help professional teams understand the role of coping responses and tolerance to stressors, which are so common and impactful during the Covid-19 pandemic.

In terms of coping strategies, as expected, control was associated with lower PS levels. Men who described themselves as able to face the challenges and analyze the problems associated with stressful pandemic events, for instance, were more effective at managing their stress levels. This is similar to findings reported in other studies^(27)^ regarding an individual’s sense of control associated with lower stress levels and better mental health outcomes in general, and in the Covid-19 context, in particular. However, unexpectedly, social support alone did not directly contribute to the prediction of PS in the present study.

Contrary to expectations, isolation strategies predicted lower PS levels in the present study. Overall, this strategy has been associated with harmful health outcomes^(17)^. However, researchers must consider the dynamic nature of coping strategies. For instance, in the present pandemic context, in which shelter-in-place measures have been strongly recommended by health experts, isolating (i.e., physical distancing) might assume a protective and adaptive role, lowering individual PS levels associated with the risks of Covid-19 exposure and contamination. In this sense, the present study contributes to a dynamic and contextual understanding of coping strategies. Finally, refusal was associated with greater PS levels. This is not surprising because the literature has consistently shown that individuals who avoid thinking and dealing with their reality present higher levels of mental suffering^(18,28)^. Therefore, it becomes urgent to accommodate such evidence in nursing education and research to overcome existing gaps in daily practice in terms of extended and integral male health care.

The moderation analysis corroborated the hypotheses that coping strategies, specifically isolation and social support, lessened the relationship between IU and PS in men. However, this might happen for different reasons. Isolation might have dampened the relationship between IU and PS by decreasing the individual’s likelihood of exposure to the contaminating agent (i.e., SARS-CoV-19) and its associated adverse health outcomes. Social support, in contrast, might have provided men with a sense of connection and emotion sharing that has been consistently associated with positive mental health outcomes^(18,19)^. In this regard, researchers have called attention to the conceptual and practical distinction between physical distancing and social distancing^(28,29)^.

This issue highlights the need to consider social support through different means (e.g., social networks, social media, online social support groups, telemedicine, and telemental health care). However, these strategies should still respect shelter- in-place measures while acknowledging that social support is a protective factor of an individual’s mental health during the pandemic. Our study findings seem to contribute to this discussion by offering empirical evidence of the benefits of social support in the context of increasing uncertainty and stress. In addition, our findings raise the need to adopt theoretical models in nursing and health, which can explain and improve clinical and managerial practice with a focus on social support for the male public.

Taken together, the present study’s findings contribute to the growing literature on mental health issues intensified by the Covid-19 pandemic in different contexts and diverse populations. More specifically, for clinical nursing practice, this means that professionals should consider this population’s indicators of psychological distress, such as IU and PS. Furthermore, the potential benefits of coping strategies should also be assessed and incorporated into different practice domains, for instance, clinical, community, and policy-making. Men have been more vulnerable to Covid-19 contagion^(28)^ and more likely to commit suicide^(28,29)^, which can be predicted by mental health disorders and socioeconomic factors associated with the pandemic^(27)^. As such, men’s mental health issues should be taken seriously in this context. This study advances our understanding of factors that might impair men’s mental health, such as high levels of IU, and those that might buffer their impact, such as coping strategies (e.g., isolation and social support). In this sense, it is crucial to strengthen health care practices by fostering competencies^(29)^ and skills specific to nursing in men’s health and by producing social, educational, and care technologies geared towards this population^(30)^.

However, these findings might be considered cautiously. This is a cross-sectional study, which limits our ability to establish causal relations between its variables. It has also been conducted online using a snowball sampling technique and focused on intra-individual variables. Furthermore, we have not deepened the analysis of other variables, such as sexual orientation and gender identity. It is believed that by adopting this recruitment technique, the sample consisted primarily of men who self- identified as non-heterosexual. Therefore, the limitation of generalization and analytical scope and the need for future investigations that analyze other individual specificities of men and macro-structural dimensions, such as social class, work, and vulnerabilities, should be considered. These factors might help further our understanding of the contextual and dynamic nature of coping strategies. Also, studies might examine other intrapersonal resources that might exacerbate or dampen the relationship between IU and mental health outcomes, such as personality traits (e.g., neuroticism and openness to experience). Given current knowledge, the nursing and health team will institute more effective interventions, which address the health needs and specificities of men exposed to stressors, establish focal care plans, expand the intervention repertoire in mental health. Thus, they can coordinate strategic and contingent actions, which are essential to the pandemic context.

Regarding public policies related to mental health, interventions based on social support in varied forms seem to be a promising way of promoting positive outcomes during the pandemic. In addition, however, physical distancing strategies that seem effective in avoiding the spread of the virus should also be considered. Finally, it is noteworthy that this study contributes to nursing and mental health, as it seeks to strengthen the production of scientific knowledge aimed at male health, mental health, and pandemics. In addition, it contributes to implementing the National Policy for Integral Attention to Men’s Health and highlights findings that can guide professional practice and decision-making policies at local and global levels.

## CONCLUSION

The main findings of this study support the empirical literature on the buffering effects coping strategies have on the relationship between individual variables and adverse mental health outcomes. More specifically, they reveal that IU is a vulnerability factor for psychological disorders (i.e., stress) in the Covid-19 context and that coping strategies such as control, isolation, and social support offer some protection against its harmful effects on men’s mental health. Therefore, health care providers might seriously consider these results when tailoring interventions at the individual and collective levels during the Covid-19 pandemic.
